# Pairwise Neural Networks for Ranking Molecular Structures Based on Properties

**DOI:** 10.1021/acsomega.6c00717

**Published:** 2026-02-12

**Authors:** Renato Frazzato Viana, Juarez L. F. Da Silva, Luis G. Dias, Ronaldo C. Prati

**Affiliations:** † Center of Mathematics, Computation and Cognition, 74362 Federal University of ABC, Av. dos Estados, 5001, Santo André, São Paulo 09210-580, Brazil; ‡ São Carlos Institute of Chemistry, University of São Paulo, Av. Trabalhador São-Carlense 400, São Carlos, São Paulo 13560-970, Brazil; § Chemistry Department, 124588 FFCLRP, University of São Paulo, Ribeirão Preto, São Paulo 14040-901, Brasil

## Abstract

The rapid discovery and design of new molecules drive innovation in science and technology, advancing energy storage, catalysis, and drug development. Traditionally, exploring chemical space involves costly quantum-chemical calculations or slow experimental screening, which limits the speed of identifying promising candidates. Machine learning has emerged as a groundbreaking approach to accelerate molecular discovery by predicting key properties directly from molecular structures. Moreover, in many cases, if we can rank molecular structures, it is not necessary to know the exact value of a molecular property. In other words, a ranker model can be useful for molecular screening. In this work, we develop a deep learning model to rank molecular structures using a siamese network approach and pairwise learning to learn the ranking. According to different properties of the QM7x and QO2Mol data sets, the results show that the performance of the learn-to-rank Siamese architecture outperforms standard pointwise regression for predicting absolute energetic properties, such as total and orbital energies, while traditional pointwise regression remains effective for derived (e.g., HOMO–LUMO gap) or nonenergy properties (e.g., dipole moment). To further validate the robustness of the proposed framework, we extended our evaluation to include the Uni-Mol molecular representation model. Experiments with Uni-Mol *V*1 and *V*2 across various model sizes (84 M to 1.1 B parameters) confirm that the pairwise learning-to-rank objective consistently outperforms standard pointwise regression, even when using highly expressive pretrained Transformer backbones.

## Graphical abstract

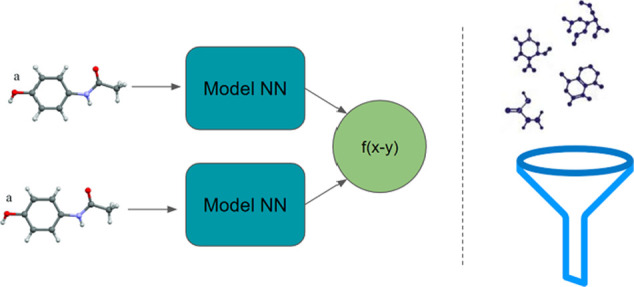

## 1 Introduction

In recent years, deep learning methodologies^1^ have achieved considerable success in diverse domains, notably contributing to advances in chemistry and materials science.^2^ In particular, quantum chemistry is an area where machine learning is making substantial progress, including applications for property prediction, drug discovery, and the innovation of new materials.^3^

Although quantum mechanics and physical laws theoretically enable the calculation of molecular properties using first-principles methodologies, such as density functional theory (DFT) calculations,^4^ the computational cost of solving the Kohn–Sham equations often makes them difficult to apply to all materials. An alternative approach is the use of machine learning techniques, especially deep learning, which produce promising results in predicting molecular and material properties.^2,5–9^ Once trained, a machine learning model can predict the properties of new data points outside the training set, often with high accuracy and at a lower computational cost than first-principles DFT methods.

Various deep learning architectures have been proposed to handle different types of data, such as images, texts, and graphs. In the context of chemistry and materials science, graph neural networks (GNNs) are particularly valuable as they can directly encode the three-dimensional structures of molecules as graphs. GNNs utilize a neighborhood aggregation approach in which the feature vector of a node is derived by progressively combining and modifying the feature vectors of its neighboring nodes.

Xu et al. explored the expressive power of GNNs and proposed an approach that is robust to graph isomorphism. They introduced a theoretical framework for analyzing the ability of GNN to capture different graph structures, characterizing the discriminative power of popular GNN variants such as graph-convolutional networks and GraphSAGE. Their findings show that these models cannot distinguish certain simple graph structures. Furthermore, they proposed an architecture that, according to their analysis, is the most expressive among graph neural networks. They also claim that their proposed architecture is as powerful as the Weissfeiler–Lehman graph isomorphism test.^10^

Zhang et al. developed a new graph grouping layer called SortPooling and applied it to graph classification. In their architecture, the authors use graph convolution to extract node features, and after several graph convolution layers, they used traditional 1-D convolutional layers on top of the graph node feature matrix. However, to apply traditional 1-D convolutional layers, a consistent representation of the graph is required, which means that permutations in node labeling should not alter the final representation of the molecular features. Essentially, after applying several graph convolutional layers, the authors concatenated the obtained features of the nodes and sorted the matrix according to the values in the last column.^11^ For additional details and discussions on predicting molecular properties with GNN, various review articles are available.^2,6,12–14^

Beyond GNNs, the rapid advancement of Natural Language Processing has inspired a new wave of molecular representation learning based on the Transformer architecture. Models such as Uni-Mol^15,16^ leverage self-attention mechanisms to capture long-range dependencies and complex 3D geometric features that might be challenging for standard message-passing frameworks. By pretraining on massive data sets of molecular conformations, these architectures have established new state-of-the-art benchmarks for various molecular property prediction tasks.

Although the adoption of machine learning is extensive, it is crucial to acknowledge the absence of theoretical guaranties pertaining to the precision of its predictions. Even models with high accuracy may demonstrate biases that result in systemic errors^17,18^ This constraint has spurred the advancement of hybrid models^19,20^ that integrate machine learning predictions with first-principles calculations to mitigate uncertainty in critical areas.

However, for the rapid screening of specific molecular property configurations, a family of machine learning algorithms remains underutilized. This family of algorithms is related to the task of learning-to-rank,^21^ where the training data consist of (partially) ordered lists of elements ranked according to specific criteria. Notable recent applications of this approach are related to the ranking of chemical structures in drug discovery^22^ and for virtual screening based on ligands.^23^ More recently, the ConfRank^24^ and ConfRank+^25^ models have successfully applied a pairwise training method for the energetic ranking of molecular conformers, with the latter demonstrating particular strength in handling charged molecules.

Given a new list of candidates, a learned ranking model will suggest a permutation of this list that sorts (or ranks) its elements similarly to the training data. For example, in quantum chemistry data, it is possible to train a model using a molecule with various structures classified by their energies or other properties. Then, given a new list of distinct configurations of a new molecule, whose ordering is unknown, the ranking model can predict an appropriate ranking for this list. This approach can potentially speed up first-principles calculations by, for instance, applying DFT only to the top-ranked items to find the desired state configuration instead of performing DFT for all molecules.

Learning-to-rank offers several key benefits. First, it is not essential to develop a highly accurate predictive model, as long as the ranking algorithm can effectively place the most relevant items at the top of the list. Furthermore, since we are primarily concerned with the relative position of the predictions rather than their actual values, there is no need to create methods to quantify the uncertainty of the prediction, which is a challenging task in machine learning. Finally, in some cases, learning-to-rank can be computationally more efficient than training a full predictive model.

In learning-to-rank, we are not aiming to predict the exact molecular property. Instead, our objective is to learn the order relation among the target properties. As explained earlier, an application of this approach lies in molecular screening—for instance, when a chemist seeks to identify the structure with the lowest energy among a set of candidate structures. In such cases, the absolute value of the energy is less relevant, since the chemist is primarily interested in finding the configuration with the minimum energy level. Therefore, a Siamese architecture is a natural choice for pairwise learning-to-rank. In this work, we develop a deep learning architecture based on a Siamese neural network to predict the ranking of molecular properties. Our model can rank molecular structures and can be applied to molecular screening. We trained the model to differentiate pairs of molecular structures using a pairwise training approach and compared the results with a model trained using a pointwise approach.

Furthermore, despite the expressive power of these pretrained Transformer models, their application has largely focused on absolute property prediction (classification or regression). It remains an open question whether the specific task of ranking molecular structures—crucial for efficient screening—can be further improved by shifting the training objective from pointwise regression to a pairwise learning-to-rank framework, or if the pretrained features are already robust enough to handle ranking tasks without specific adaptation. Addressing this question is essential for understanding whether the benefits of pairwise ranking are universal or specific to certain architectures, such as GNNs.

Although our methodology shares conceptual foundations with recent ranking methods such as ConfRank and ConfRank+, our work introduces a distinct approach in several key areas. First, our model is trained and validated on the comprehensive QM7x and QO2Mol data sets, which focus on a diverse set of molecules in both equilibrium and nonequilibrium configurations, whereas ConfRank and ConfRank+ were specifically engineered to address the challenge of ranking charged conformers. Second, we use a Siamese neural network architecture, inspired by Koppel et al.,^26^ and the results demonstrate promising performance for the learning-to-rank task for different properties. Furthermore, our objective is to establish a general ranking framework for screening the varied structures, distinguishing it from the shared focus of both ConfRank and ConfRank+ on the more specific task of ranking conformational ensembles. The Siamese model provides significant improvements in some cases while yielding results comparable to those of the point-wise approach in others.

## 2 Machine Learning Fundamentals and Methodology

This section provides an overview of the literature on GNNs for molecular data. In addition, we describe the architecture we developed, how it was adapted to learn rankings, and the data used for the evaluation.

### 2.1 Neural Graphs Fingerprints

Gilmer et al.^8^ present a general deep learning framework called Neural Message Passing. This framework provides a general overview of the models applied to molecular data. The forward pass in this framework is divided into two phases, where the first phase is called message passing, and the second is the read-out phase. The message passing phase runs for *T* times and is defined in terms of two learned differentiable functions: the message passing function *M*_*t*_ and the update function *U*_*t*_. These functions define how information is passed between nodes (atoms) in a molecular graph and how each node updates its internal representation.

The message function *M*_*t*_ determines how messages are constructed in time step *t* based on the features of the nodes and edges. This is the mechanism through which the nodes communicate with each other. Let *h*_*i*_^*t*^ represent the hidden state (or node feature) of a node *i* at time step *t*, and let *e*_*i*_^*j*^ represent the edge feature between nodes *i* and *j* (for example, the bond between two atoms). The message function (expression [Disp-formula eq1]) is designed to compute the message that a node *i* receives from its neighboring node *j*. This is generally denoted as

$$m_{ij}^{t+1}=M_{t}(h_{i}^{t},h_{j}^{t},e_{i}^{j})$$ 1

where *m*_*ij*_^*t*+1^ is the message sent from node *j* to node *i* at time *t* + 1. The message function *M*_*t*_ can take various forms depending on the specific implementation. Typically, this involves neural network layers, such as fully connected layers that combine the features of the neighboring nodes and the edge that connects them.

The update function *U*_*t*_ is used to update the hidden state of node *i* based on the incoming messages from its neighbors and its current state. Once all the messages from the neighboring nodes have been collected, the node *i* aggregates them, usually by summing or averaging, to produce an aggregate message:

$$m_{i}^{t+1}=\sum_{j\in N(i)}m_{ij}^{t+1}$$ 2

where *N*(*i*) represents the neighbors of the node *i*. The node’s hidden state is then updated using the update function *U*_*t*_, which takes the node’s current hidden state *h*_*i*_^*t*^ and the aggregated message *m*_*i*_^*t*+1^ as inputs:

$$h_{i}^{t+1}=U_{t}\left(h_{i}^{t},m_{i}^{t+1}\right)$$ 3

The update function *U* ^*t*^ is usually implemented as a neural network layer, such as a GRU (Gated Recurrent Unit) or another type of recurrent unit that allows the node to retain memory across time steps. This enables the model to capture more complex dependencies between atoms over multiple message-passing iterations.

Another approach, described in Algorithm 1,^27^ generates a molecular fingerprint, a vector of fixed-length real values that can be used as a representation of features in various downstream tasks. This fingerprint is learned directly from the molecular graph structure and can be used in tasks such as molecular property prediction. The algorithm applies the neural message-passing principle, as outlined previously, to iteratively update the atomic representations by exchanging information between neighboring atoms in the molecule.
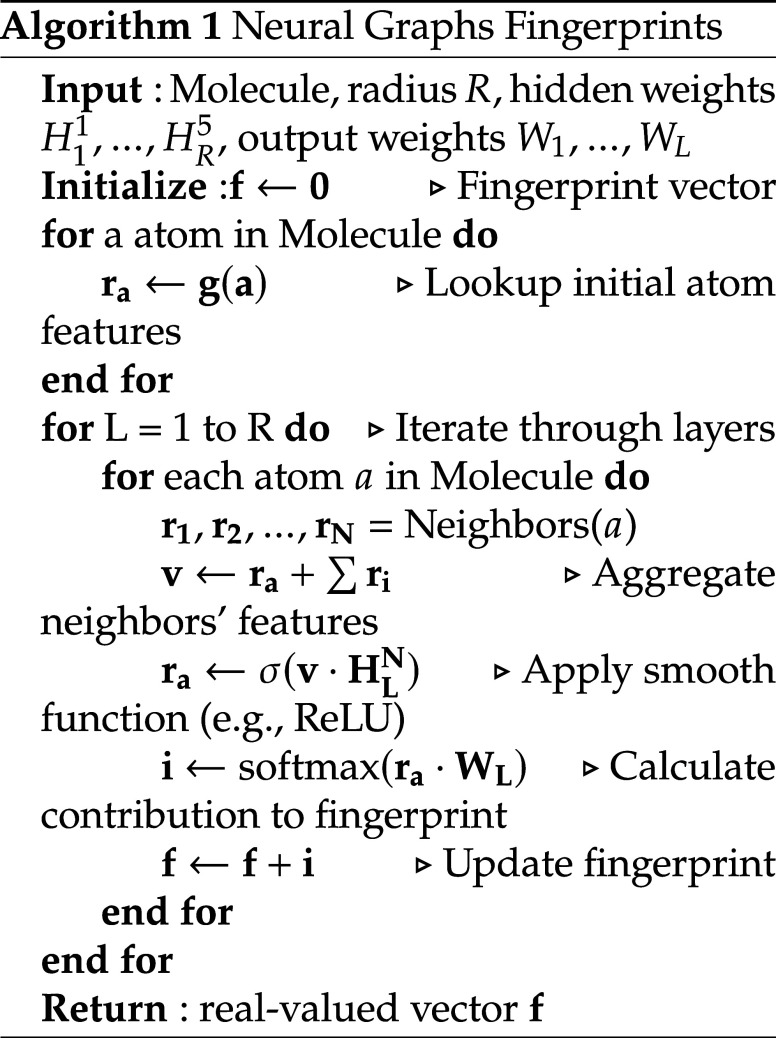


At each layer *L*, the Algorithm 1 iterates on all atoms of the molecule, combining the characteristics of each atom with those of its neighboring atoms. This produces a refined representation of each atom. The molecular fingerprint is obtained by summing the contributions of each atom across layers, ensuring that the resulting representation is invariant to the permutation of the graph’s vertices (atoms). This property is crucial because the underlying graph of the molecular structure can have different node orderings, but the fingerprint remains consistent.

### 2.2 SchNet Architecture

SchNet^28^ is a tailored neural network designed to learn the representation of atomistic systems. SchNet has achieved exceptional results in the prediction of quantum properties, including molecular energies and atomic forces, by learning continuous, atomic-level representations of molecules. The architecture of SchNet integrates several essential invariances pertinent to quantum chemical data. Its representation remains invariant to the indexing of atoms and translational transformations, thereby ensuring that the molecular representations are independent of the sequence in which atoms are indexed and their absolute spatial positions. Furthermore, considering the interatomic distances, SchNet achieves rotational invariance, ensuring that the predictive results are consistent regardless of molecular orientation.^14,28^

In SchNet, a molecule is composed of a collection of *n* atoms, each characterized by nuclear charges *Z* = (*Z*_1_,..., *Z*_*n*_) and specific atomic positions *R* = (**r**_1_,..., **r**_*n*_). Within each layer *l*, these atoms are represented by features *X* ^*l*^ = (*x*_1_^*l*^,..., *x*_*n*_^*l*^), denoted by $x_{1}^{l}\in R^{F}$ , where *F* represents the number of features, and *n* represents the number of atoms. The initial representation of the atom *i* is a randomly initialized embedding vector **a**_*Z*_*i*__ that depends on its atomic number *Z*_*i*_, as shown in expression [Disp-formula eq4]. These embeddings are learned during the training process.

$$x_{i}^{0}=a_{Z_{i}}$$ 4

The next component is the atom-wise layer, which is essentially a fully connected or dense layer (multilayer perceptron or MLP). In this layer, the features of each atom are updated independently. Consider the atom characteristics **x**_*i*_^*l*+1^ as row vectors, as shown in Expression [Disp-formula eq5].

$$x_{i}^{l+1}=x_{i}^{l}\cdot W^{l}+b^{l}$$ 5

In expression [Disp-formula eq5], *W* ^*l*^ and *b* ^*l*^ represent the weights of the hidden layers and the biases of the neurons, respectively. In particular, the weights within each layer are shared across all atoms. The last component of SchNet is the interaction layer. This layer is responsible for the interaction of each atom with its surrounding atoms. The interaction is based on the features of the atoms and the distances between pairs of atoms. Consider that **v** *_i_^l^* is the vector with information from the neighborhood of atom *i*, the characteristic vector of atom *i* is updated according to the residual connection inspired by ResNet.^29^

$$x_{i}^{l+1}=x_{i}^{l}+v_{i}^{l}$$ 6

The calculation of **v**_*i*_^*l*^ begins with an MLP applied to the atom’s features. This is then followed by a continuous filter convolution, which allows the atom *i* to interact with all other atoms in the molecule, aggregating their contributions as shown in expression [Disp-formula eq7].

$$\sum_{j\neq i}\mathrm{MLP}[x_{j}^{l}]⊙\sigma (\mathrm{MLP}[\sigma (\mathrm{MLP}[d_{ij}])])$$ 7

The vector **d**_*ij*_ is obtained through transformations of the Euclidean distance (*d*_ij_^*^) using the radial basis function, which is expressed by expression [Disp-formula eq8].

$$d_{ijk}=\exp(-\gamma (d_{ij}^{*}-\mu_{k}{)}^{2})$$ 8

After the calculations described in expression [Disp-formula eq7], **v**_*i*_^*l*^ is obtained by applying one atom-wise layer, an activation function, and another atom-wise layer. Then, the value **v**_*i*_^*l*^ is added to **x**_*i*_^*l*^. Figure 2 shows the model architecture, and we can see the details of each module. The function σ is a softplus-shifted function. Figure 2a provides an overview of SchNet, illustrating the integration of all components of the model. Figure 2b shows the interaction block, which, in combination with Figure 2c, facilitates interactions between molecular atoms. The interaction block (Figure 2b) receives the output of block (c), applies two dense layer transformations, and sums the result with its input. Block (c) computes the contribution of each atom *j* to atom *i*, expands the Euclidean distance using a radial basis function, and applies two dense layers on top of the RBF expansion.

**Figure 1:**
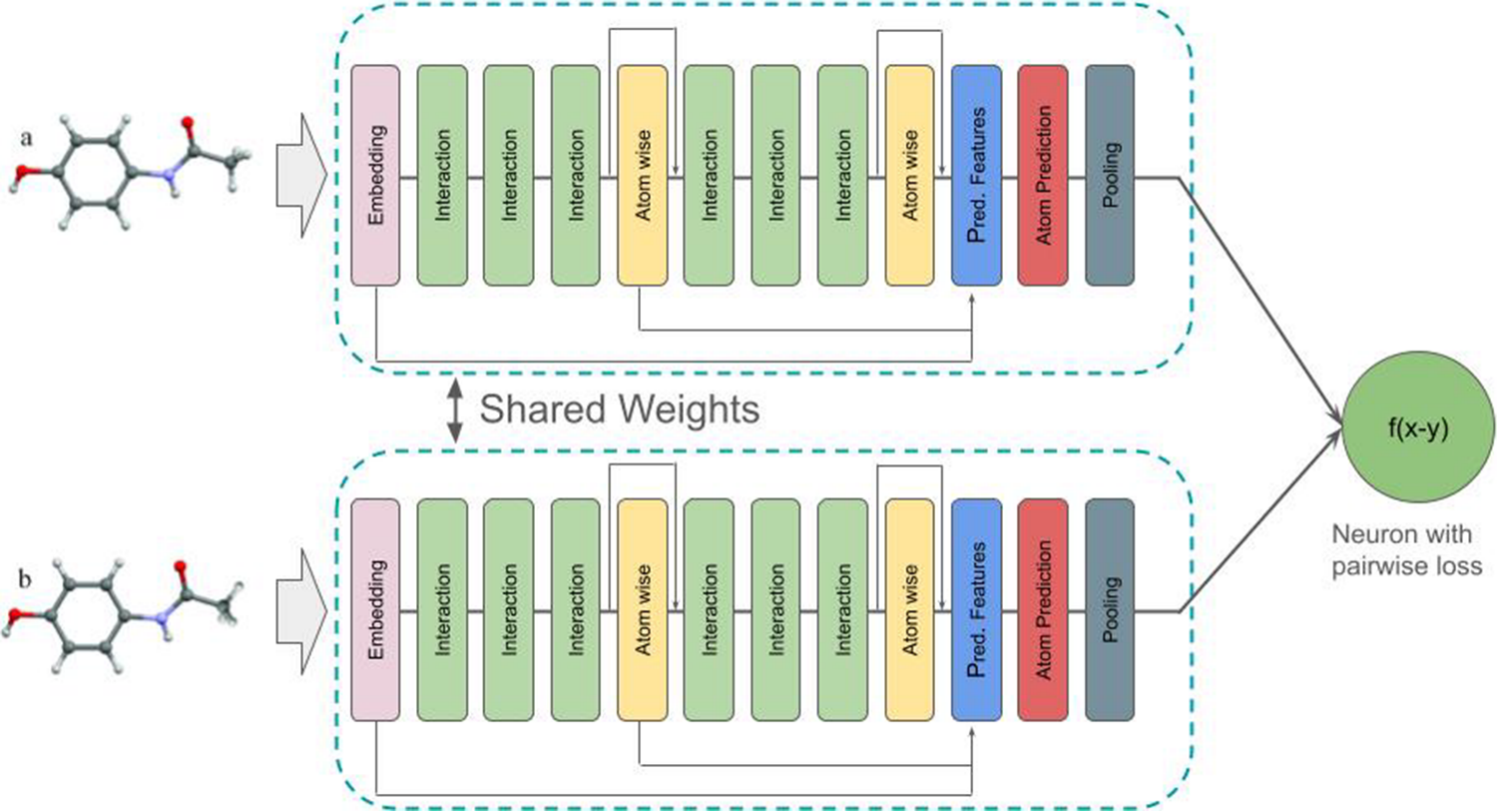
Ranking architecture overview. The two neural networks have shared weights and the outputs are combined in order to learn the ranking.

**Figure 2:**
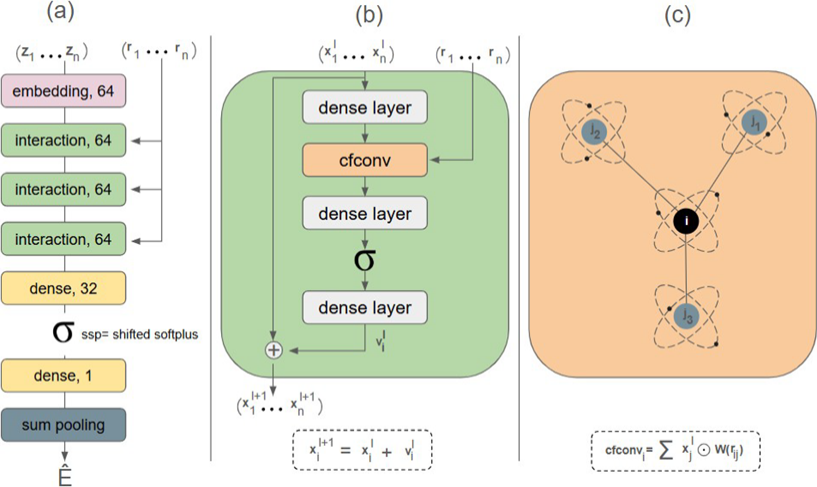
(a) SchNet architecture overview. (b) Interaction block. (c) Continuous filter convolution block. The shifted softplus is defined as ssp­(*x*) = ln­(0.5*e* ^*x*^ + 0.5). Adapted from ref 28. Copyright 2017 The Authors.

### 2.3 3D Molecular Features Based on Transformers: Uni-Mol

While Graph Neural Networks (GNNs) such as SchNet rely on message-passing mechanisms to update atomic representations based on local neighborhoods, recent advancements have introduced Transformer-based architectures for molecular modeling. Uni-Mol^15,16^ is a state-of-the-art 3D molecular representation learning framework that takes advantage of the self-attention mechanism^30^ to capture global interactions between atoms.

Unlike 1D string representations (SMILES) or 2D topological graphs, Uni-Mol explicitly incorporates 3D spatial information. Crucially, the architecture is designed to respect the physical symmetries of 3D space (specifically, the SE(3) group). This means that the model processes molecular coordinates in a way that is invariant to translations and rotations, ensuring that the learned representation—and consequently the predicted property—remains consistent regardless of the molecule’s orientation or position in the simulation box.

The model is pretrained on large-scale data sets (containing millions of conformations) using self-supervised learning tasks, such as predicting masked atoms and denoising 3D coordinates. This pretraining strategy allows Uni-Mol to learn rich, transferable features that capture complex geometric and electronic properties. In the context of our work, Uni-Mol serves as a powerful feature extractor, providing fixed-length molecular embeddings that can be fed into downstream task-specific heads, such as the ranking objectives explored in this study. Utilizing these pretrained representations is highly efficient, as it bypasses the extensive computational cost of training the deep feature extraction layers from scratch. We utilized both version 1 (V1) and the improved version 2 (V2) of the framework to evaluate the impact of using robust pretrained representations for the ranking task. We note that while Uni-Mol V1 is designed to encode the specific geometry of the input conformation, Uni-Mol V2 (Uni-Mol+) explicitly optimizes structures toward their equilibrium state. This refinement process may inadvertently smooth out the distinct high-energy geometric features that are essential for distinguishing between conformers in a ranking task.

## 3 Materials and Methods

SchNet is a distance-based model that has become a benchmark in the literature; it relies solely on interatomic distances and atomic numbers to learn atomic representations. This makes it a simpler approach compared to architectures such as PaiNN^31^ and DimeNet,^32^ which incorporate atomic positions and spherical harmonics to extract directional information. To validate our ranking framework, we implemented a model inspired by SchNet, as it provides a good compromise between a well-established, computationally cheaper benchmark and more demanding architectures. However, architectures such as PaiNN^31^ and DimeNet,^32^ as well as other powerful alternatives such as MACE,^33^ can also be used.

The architecture of our model follows an idea similar to that of Schnet, including an embedding layer for the atoms, an atom-wise layer, and interaction layers. However, there are some differences. The interaction layer is described by expression [Disp-formula eq9]. The proposed model has a simpler design. Instead of creating modules of continuous filter convolutions and interactions, we adopt the approach of Gilmer,^8^ in which the interaction module defines the process of passing messages and updating features for each atom. We also include a decay function, which, together with the expansion of the radial basis, serves to weight the relevance of atom *j* in atom *i*.

Furthermore, it is well-known that adding many layers to a model can lead to issues such as gradient vanishing, which prolongs the training time. To address this, we include a prediction layer that aggregates features from different depths of the model, as shown in the expression [Disp-formula eq11].

$$\begin{array}{ll} m_{i}^{t+1} & =\sum_{j\neq i}\phi (d_{ij})\cdot x_{j}^{t}⊙\mathrm{MLP}[\sigma (\mathrm{MLP}[d_{ij}])]\\ x_{i}^{t+1} & =x_{i}^{t}+\mathrm{MLP}[\sigma (\mathrm{MLP}[m_{i}^{t+1}])] \end{array}$$ 9

The decay function ϕ­(*d*_*ij*_)^6^ is a distance-dependent factor that decreases as the distance *d*_*ij*_ between the atoms increases, as defined by expression [Disp-formula eq10]. The cutoff distance *r*_cut_ is a hyperparameter learned during training. Additionally, the dimension of the atoms’ distance radial basis function expansion is set to 300, with a scaling factor γ = −1.0, and the *k*-dependent parameters μ_*k*_ are uniformly distributed between 0 and 30 with a step size of 0.1. Our architecture is shown in Figure 3.

$$\phi (d_{ij})=1-6{\left(\frac{d_{ij}}{r_{\mathrm{cut}}}\right)}^{5}+15{\left(\frac{d_{ij}}{r_{\mathrm{cut}}}\right)}^{4}-10{\left(\frac{d_{ij}}{r_{\mathrm{cut}}}\right)}^{3}$$ 10

**Figure 3:**
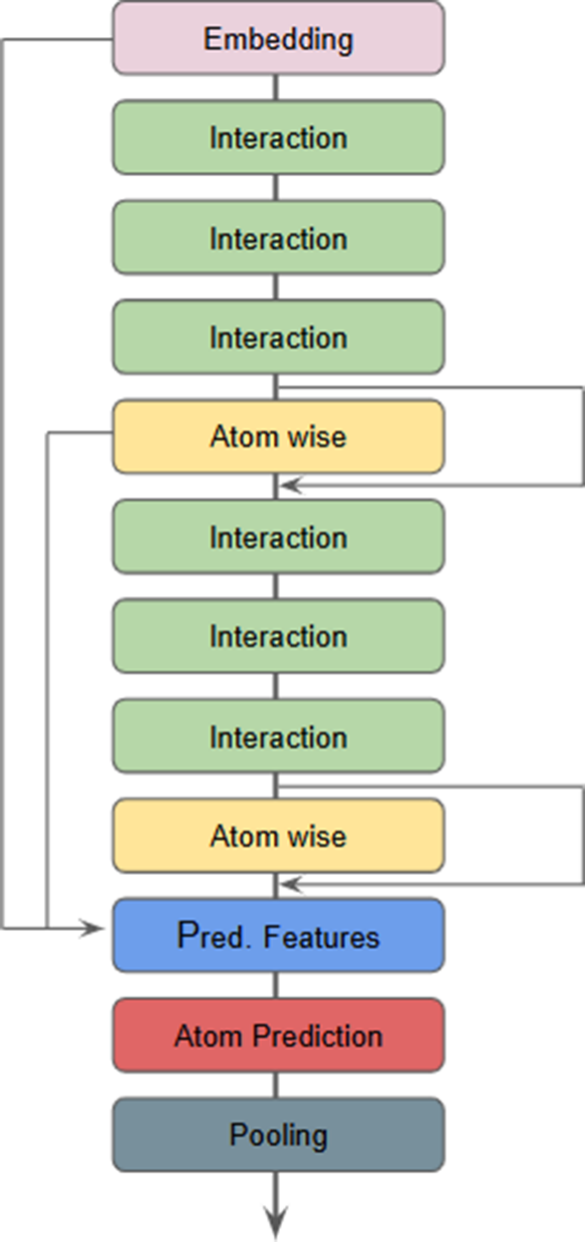
Model architecture overview with the indication of the workflow.

The prediction layer incorporates the atom feature through a skip connection, combining the outputs from various parts of the model. As depicted in Figure 3, this layer merges the embedding with the output of two distinct layers atom-wise. This approach facilitates the flow of gradients throughout the model, enhancing the learning process.

$$p_{i}=u⊙E_{i}+w_{1}⊙\mathrm{atw}1_{i}+w_{2}⊙\mathrm{atw}2_{i}$$ 11

### 3.1 Deep Learning for Molecular Ranking: Pairwise and Pointwise Strategies

The learning-to-rank problem involves identifying the most relevant order of items based on a specific criterion. Our research applies this concept to learn to rank various nonequilibrium molecular structures by their properties, such as atomization energy, to find an ordering consistent with the inherent ordering of the structure’s properties. DirectRanker, which was introduced by Köppel et al.^26^ is a neural network architecture designed for learning ranking tasks that generalizes the RankNet architecture.^34^ It has several interesting properties for learning rank, such as reflexivity, antisymmetry, and transitivity, allowing for simplified training and improved performance. DirectRanker combines two identical models with shared weights, followed by a final layer that learns to rank items in a pairwise manner. This approach is visualized in Figure 4.

**Figure 4:**
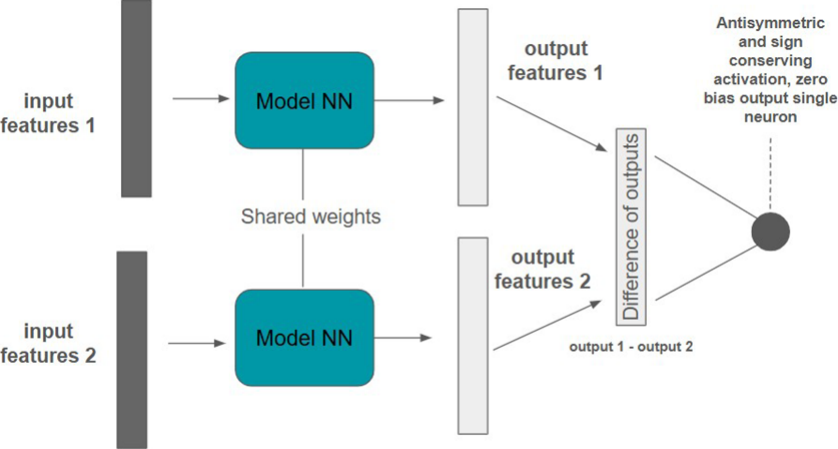
DirectRanker architecture. Adapted from ref 26. Copyright 2020 The Authors. Licensed under CC BY 4.0.

We adapt the model architecture shown in Figure 3 to tackle the ranking task. The architecture can be seen in Figure 1. Specifically, we used a pairwise approach in which the model scores pairs of molecular structures of the same molecule and calculates the differences in scores. A logistic function then converts this difference into a probability. To optimize the model, we used cross-entropy as the loss function and trained it as a binary classifier. For example, given two pairs of structures, *m*_1_ and *m*_2_, with target values *t*_1_ and *t*_2_, the model is trained to predict a label of (1, 0) if *t*_1_ > *t*_2_, (0, 1) if *t*_1_ < *t*_2_, and (0.5, 0.5) if *t*_1_ = *t*_2_.

We also investigated an alternative approach based on regression in which the model aims to predict the difference in property values between two molecular structures *m*_1_ and *m*_2_. If the predicted difference is positive, *m*_1_ is ranked higher; if the difference is negative, *m*_2_ is ranked higher. In both scenarios (predicting probabilities or differences), the final ranking of multiple structures is constructed by iteratively applying this pairwise ranking process, with the structure receiving the highest predicted probability or difference value ranked first, and so on.

The batch size for model training is 64 pairs, and the models were trained for up to 60 epochs. The learning rate is 1 × 10^–4^ and the number of neurons in the layers is kept constant at 128. The models were implemented using TensorFlow and trained using an L4 and A-100 GPU. Both pairwise approaches are compared to a traditional regression model, which is trained to predict property values on a point-wise basis. In this case, the structures of the molecules are ranked according to their predicted property values.

### 3.2 Ranking with Pretrained Representations

To evaluate the generality of our pairwise ranking framework beyond the SchNet architecture, we utilized features extracted from the Uni-Mol 3D molecular pretraining framework based on the Transformer architecture described in Section 2.3. Since our data sets represent molecules using Cartesian coordinates, we used Uni-Mol (both V1 and V2) as a feature extractor to generate fixed-length molecular embeddings for the molecular structures.

These precomputed embeddings serve as the input for a Multi-Layer Perceptron (MLP) head (Figure 5), which consists of a hidden layer with 128 neurons, a hyperbolic tangent activation function, and a second hidden layer of 64 neurons. This model was trained using the same pairwise (entropy and squared loss) and pointwise objectives described in Section 3.1. We evaluated performance across five model sizes (84 M to 1.1 B parameters) to assess scalability. This setup allows us to decouple the impact of the training objective from the model architecture, determining whether the pairwise ranking benefit persists with state-of-the-art representations.

**Figure 5:**
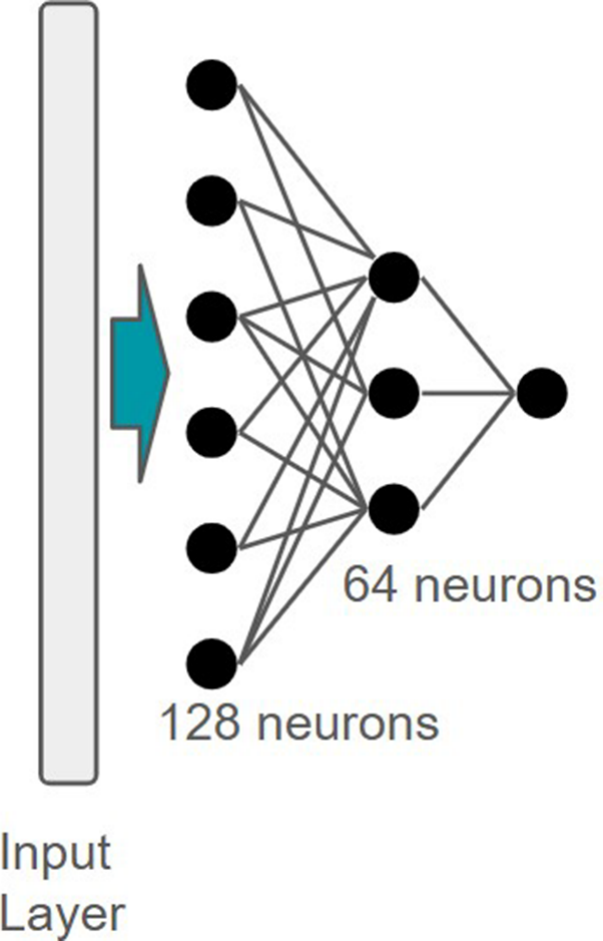
Multi Layer Perceptron architecture trained with unimol features.

### 3.3 Molecular Data Sets

A key requirement for our study was the use of data sets containing a diverse set of geometries for each molecule. We selected benchmarks that include both the optimized equilibrium configuration and a significant number of nonequilibrium structures, which are essential for a robust ranking analysis.

Common choices for data splits in training and testing are 80–20%, 90–10%, and 95–5%. Without loss of generality, we separated 5% of the molecules for testing and 95% for training. Given the large size of the data sets used, the 5% test partition still constitutes a large and diverse set of thousands of molecular structures, ensuring that our performance evaluation is statistically significant and reliable. The division was performed at the molecular level. This method ensures that if a molecule is selected for the training set, all of its corresponding configurations (i.e., its equilibrium and nonequilibrium structures) are kept in the training set, and the same applies to the test set. This approach prevents data leakage and guaranties a fair evaluation of the ability of the model to rank conformers of unseen molecules. The primary goal is to assess whether our pairwise training approach, illustrated in Figure 4, outperforms a traditional pointwise regression model on this ranking task.

#### 3.3.1 QM7x

We use the QM7x data set,^35^ a widely used benchmark in machine learning for tasks related to molecular properties and quantum chemistry. QM7x comprises equilibrium and nonequilibrium structures of small organic molecules, including constitutional/structural isomers and stereoisomers, e.g., enantiomers and diastereomers (including cis-/trans- and conformational isomers), with up to seven non-hydrogen atoms, reaching a total of 6950 molecules and approximately 4.2 × 10^6^ structures, along with their corresponding properties, including ground state and response quantities. For each equilibrium structure, 100 nonequilibrium counterparts are available.

We selected several properties available in the QM7x data set to evaluate the model, and the quantities we selected have a wide range of applications. For example, homo and lumo energies determine the ability of a molecule to donate or accept electrons, influencing its electronic and optical properties. The homolumo gap is directly related to molecular stability and conductivity, making it critical for the design of organic semiconductors, photovoltaics, and optoelectronic materials. Similarly, the dipole moment reflects the charge distribution and molecular polarity, which play a significant role in drug solubility, intermolecular interactions, and material design. Kinetic and exchange energy provide deeper insights into quantum mechanical behavior and electronic structure, aiding in the refinement of density functional theory (DFT) methods. The dispersion energy is particularly relevant for understanding weak intermolecular forces, which are critical in biological systems (e.g., protein–ligand binding) and nanomaterials. Furthermore, the PBE0 energy, a hybrid DFT functional result, improves accuracy in predicting total molecular energy, while the atomization energy reveals the strength of molecular bonds, helping to calculate thermodynamics and reaction energy. Together, these properties serve as essential descriptors in computational chemistry, enabling the rational design of novel molecules and materials with tailored properties.^36,37^ For the sake of notation, we will use the PBE0 energy to denote the total energy of the system calculated using this PBE0 functional in the results section.

#### 3.3.2 QO2Mol

The Quantum Open Organic Molecular (QO2Mol) database is a large-scale quantum chemistry data set. This database comprises 120.000 organic molecules and approximately 20 × 10^6^ conformers, encompassing 10 different elements (C, H, O, N, S, P, F, Cl, Br, I), with heavy atom counts exceeding 40.^38^ The QO2Mol data set provides molecular structures grouped by InChIKey, a unique identifier for each molecule. We used only parts A and B of the data set, as part C contains single-configuration only entries and was therefore excluded. Due to computational constraints, we trained our models on a representative sample of the data. On this final data set, we compare the ranking performance of our proposed pairwise and pointwise models against the SchNet benchmark.

## 4 Results and Discussion

In order to evaluate the ability of the pairwise approach for ranked molecular structures, we performed a comparison using ranking performance metrics. We compared the pairwise ranking model with the pointwise model, i.e., the pairwise model architecture is presented in Figure 4 where the nn1 and nn2 rectangles represent the architecture depicted in Figure 3 and the pointwise model represents the model shown in Figure 3. Moreover, we also used the SchNet model using the Python library SchNetPack^39,40^ as a benchmark. This section presents and analyzes the results by comparing true and predicted rankings. The true ranking is determined by sorting molecular structures according to their actual property values and tracking their corresponding positions. In contrast, the predicted ranking reflects the model’s output, as previously described. We report average values among all the molecules in the test set.

We evaluated our proposed model using four metrics. Mean Absolute Error (MAE) for rankings, Spearman correlation, normalized Discounted Cumulative Gain (nDCG), and the frequency of the best property being ranked first. The MAE for rankings measures the average absolute difference between the predicted and true rankings. The Spearman correlation assesses the agreement between the true and predicted rankings, with a high correlation coefficient indicating a strong positive relationship. nDCG evaluates the performance of ranking models by measuring the cumulative gain of top-ranked items, taking into account their position in the ranking. The frequency of the top-ranked structure indicates the proportion of molecules, isomers, and conformers for which the model correctly ranks the molecular structures with the lowest energy or best property value as the first item. It is important to apply more than one metric to evaluate Machine Learning models because each metric captures different features of the data; moreover, when several performance metrics are used, it is possible to assess the robustness of the model performance.

The results are presented for both pairwise approaches, that is, the probabilistic prediction of the model trained using cross-entropy loss and the pairwise model trained using regression squared loss. In addition, a confidence interval of 95% is provided for each of the four metrics. We report the mean value of each performance metric and its standard deviation achieved by our ranking architecture. Performance for point-wise model training is also presented (point-wise column).

### 4.1 Evaluation Using the QM7x Data Set

We evaluated the models in 10 different molecular properties available in QM7x, such as the energy of the homo, lumo, and homolumo gap (Δ). Understanding molecular properties, such as homo and lumo energy levels, the homolumo gap, the dipole moment, kinetic and dispersion energy, exchange energy, PBE0 energy, and atomization energy, is fundamental to advancing research in chemistry, materials science, and drug discovery. These properties provide crucial insights into molecular stability, electronic behavior, and reactivity, which are essential for designing new materials, optimizing chemical reactions, and understanding molecular interactions at a fundamental level.

#### 4.1.1 Comparative Analysis of Ranking Metrics

Table 1 presents the results for the mean absolute error (MAE) metric, where our ranking architecture outperforms the point-wise approach for five key properties: homo, PBE0 energy, kinetic, dispersion, and classical Coulomb energies. Our architecture achieves significantly lower confidence intervals for these properties, indicating improved accuracy in ranking molecules. In particular, we observe substantial advantages over the pointwise approach for the PBE0 (2.364 vs 19.557), kinetic (0.858 vs 4.091), and classical Coulomb (1.184 vs 4.135) energies. Although our architecture does not outperform the pointwise approach for lumo, atomization, and exchange energies, as well as the scaled dipole moment and homolumo gap, the differences are not substantial.

**Table 1:** Mean Absolute Error (MAE) for the Predicted Ranking of Molecular Structures^a^ ^b^ ^c^ ^d^

property	entropy loss	squared loss	pointwise
*E*_homo_	11.567 ± 0.15	12.744 ± 0.17	11.968
*E*_PBE0_	2.364 ± 0.02	1.600 ± 0.02	19.557
*E*_LUMO_	5.772 ± 0.09	6.732 ± 0.10	5.619
*E*_atom_	2.456 ± 0.02	1.894 ± 0.02	1.855
μ	11.627 ± 0.20	12.605 ± 0.21	10.480
*E*_kinetic_	0.858 ± 0.01	0.437 ± 0.01	4.091
Δ_homo–lumo_	11.276 ± 0.14	9.259 ± 0.12	9.046
*E*_dispersion_	6.013 ± 0.09	2.109 ± 0.04	8.421
*E*_coulomb_	1.184 ± 0.02	0.489 ± 0.01	4.135
*E*_exchange_	0.672 ± 0.01	0.338 ± 0.01	0.625

a Lower values indicate better ranking performance.

b Entropy loss: pairwise classifier.

c Squared loss: pairwise regression model.

d Pointwise: vanilla regression model training.

In order to provide an idea of model variability, we also compute the standard deviation for the 95% confidence interval. Table 1 shows the MAE metric for the pairwise model trained using regression (squared loss), we can see an improvement due to the change in the optimization function in the following properties: PBE0 energy, atomization energy, kinetic energy, homolumo energy gap, dispersion energy, Coulomb energy, and exchange energy. On the other hand, for the homo energy and scalar dipole moment, the squared loss function had a small decrease in performance.

Table 2 provides the nDCG metric for pairwise models trained using cross entropy and squared loss, respectively. Here, we also observe that when the loss function is changed, the performance may improve for some properties. For example, in the nDCG case, the properties PBE0 energy, lumo energy, atomization energy, kinetic energy, homolumo gap, dispersion energy, coulomb energy, and exchange energy improved when trained using squared loss. When comparing pairwise and pointwise approaches, we observe a significant improvement in ranking properties such as the PBE0 energy, kinetic energy, and dispersion energy. Consistent across multiple metrics (MAE and nDCG), the pairwise approach demonstrates a clear advantage in determining the ranking accuracy for several properties. In particular, the pairwise training approach yields superior results for at least half of the properties, with a particularly significant improvement observed in the PBE0 energy property.

**Table 2:** nDCG for the Predicted Ranking of Molecular Structures^a^ ^b^ ^c^ ^d^

property	entropy loss	squared loss	pointwise
*E*_homo_	0.687 ± 0.01	0.659 ± 0.01	0.677
*E*_PBE0_	0.875 ± 0.01	0.921 ± 0.01	0.306
*E*_LUMO_	0.912 ± 0.01	0.938 ± 0.00	0.942
*E*_atom_	0.983 ± 0.00	0.994 ± 0.00	0.993
μ	0.646 ± 0.01	0.640 ± 0.01	0.693
*E*_kinetic_	0.981 ± 0.00	0.990 ± 0.00	0.886
Δ_homo–lumo_	0.829 ± 0.01	0.902 ± 0.01	0.899
*E*_dispersion_	0.867 ± 0.01	0.968 ± 0.00	0.807
*E*_coulomb_	0.988 ± 0.00	0.994 ± 0.00	0.912
*E*_exchange_	0.985 ± 0.00	0.993 ± 0.00	0.981

a Higher values indicate better ranking performance.

b Entropy loss: pairwise classifier.

c Squared loss: pairwise regression model.

d Pointwise: vanilla regression model training.

#### 4.1.2 Lowest Energy Prediction

In materials science, stable low-energy structures determine the mechanical, electronic, and optical properties of materials, which impact their performance in applications such as semiconductors and nanotechnology. We also evaluate the models’ ability to pinpoint the structure with the lowest property value. The low-energy molecular structure tends to be more stable; thus, finding it is important.

Table 3 shows the precision of the pairwise classification model and the pairwise regression model. The column in pointwise displays the accuracy of the vanilla regression model trained in a pointwise fashion. This table presents the ratio of the molecular structures of isomers and conformers, by which the models were able to correctly identify the configuration with the lowest property value.

**Table 3:** Accuracy of the Model in Identifying the Lowest-Energy Molecular Structure^a^ ^b^ ^c^ ^d^

property	entropy loss	squared loss	pointwise
*E*_homo_	0.356	0.324	0.346
*E*_PBE0_	0.652	0.757	0.008
*E*_LUMO_	0.736	0.810	0.825
*E*_atom_	0.937	0.978	0.973
μ	0.312	0.318	0.368
*E*_kinetic_	0.930	0.962	0.668
Δ_homo–lumo_	0.576	0.726	0.717
*E*_dispersion_	0.645	0.889	0.537
*E*_coulomb_	0.958	0.979	0.737
*E*_exchange_	0.944	0.973	0.930

a Higher values indicate better performance.

b Entropy loss: pairwise classifier.

c Squared loss: pairwise regression model.

d Pointwise: vanilla regression model training.

The pairwise model trained using regression squared loss (Table 3) yielded better results compared to the model trained with entropy loss (Table 3). The only property that did not show improvement was the homo energy; compared to the pointwise model, we observed substantial improvements in PBE0 energy, kinetic energy, dispersion energy, and Coulomb energy. Once again, the pairwise approach demonstrated promising results. Even for properties where the pointwise approach performed better, the pairwise model remained competitive.

#### 4.1.3 Ranking Correlation

In this section, we evaluate the Spearman correlation; this metric can be viewed as the Pearson correlation applied to ranked data. The Spearman rank correlation coefficient is widely used in various fields to measure the strength and direction of a monotonic relationship between two ranked variables. Additionally, the Spearman rank correlation coefficient provides a robust alternative by evaluating the strength and direction of monotonic relationships, where one variable consistently increases or decreases with another, but not necessarily at a constant rate. Hence, we apply Spearman to the ranks obtained by the models and the true ranking.

Table 4 shows the results for the Spearman correlation metric for the pairwise model trained using cross-entropy and squared regression loss. Moreover, the pointwise column provides the Spearman metric for the regression model trained in a pointwise fashion. This metric corroborates the previous analysis in which our ranking architecture outperforms the pointwise approach for properties such as PBE0 energy, kinetic energy, TS dispersion energy, and classical Coulomb energy when compared to the pointwise approach. In particular, we observe substantial advantages over the point-wise approach for the PBE0 energy (0.992 vs 0.590). Apart from that, in this metric, we also observed an improvement in ranking for some properties when training the model using the squared loss function. Also, one interesting aspect is the homo property, where the model trained using cross-entropy loss provided better results; it requires further investigation to understand why the classifier performed better for homo energy.

**Table 4:** Spearman Correlation for the Predicted Ranking of Molecular Structures^a^ ^b^ ^c^ ^d^

property	entropy loss	squared loss	pointwise
*E*_homo_	0.842 ± 0.00	0.810 ± 0.00	0.831
*E*_PBE0_	0.992 ± 0.00	0.996 ± 0.00	0.590
*E*_LUMO_	0.956 ± 0.00	0.941 ± 0.00	0.958
*E*_atom_	0.992 ± 0.00	0.994 ± 0.00	0.995
μ	0.833 ± 0.01	0.805 ± 0.01	0.862
*E*_kinetic_	0.999 ± 0.00	1 ± 0.00	0.974
Δ_homo–lumo_	0.850 ± 0.00	0.893 ± 0.00	0.898
*E*_dispersion_	0.955 ± 0.00	0.994 ± 0.00	0.914
*E*_coulomb_	0.998 ± 0.00	0.999 ± 0.00	0.977
*E*_exchange_	0.999 ± 0.00	1 ± 0.00	0.999

a Higher values indicate better ranking performance.

b Entropy loss: pairwise classifier.

c Squared loss: pairwise regression model.

d Pointwise: vanilla regression model training.

MAE and Spearman correlation assess the overall consistency of the ranking across all structures. In contrast, nGCD emphasizes the accuracy of top-ranked structures, providing a more nuanced evaluation. Table 2 shows the results of the nGCD metric. As explained above, we trained the ranking model in a pairwise manner. However, instead of learning a classification task, we used a regression squared loss. In other words, considering two different molecular structures, the model is trained to learn the difference between the target properties.

Overall, the pairwise approach provided better results for PBE0 energy, kinetic energy, dispersion energy, coulomb energy, and exchange energy (5 of 10 properties we evaluated). Moreover, when training the model using squared regression loss, we observed that some improvements may occur. Furthermore, the regression pairwise approach improved the detection of the lowest energy structure for all properties except for homo. In particular, for the atomization energy and the homolumo gap, the pairwise regression model performed slightly better than the pointwise regression model in terms of the nDCG and the frequency of the structure with the minimum property ranked first.

#### 4.1.4 Comparison with Standard SchNet

To benchmark our implementation against a standard baseline, we compared it with the SchNet model available in the SchNetPack^39,40^ library.^40^ To this end, we trained a SchNet neural network and compared its results with our implementation, which corresponds to the pairwise approach. Table 5 reports the performance for *E*_PBE0_, where the pairwise ranking method achieved significantly better results, as well as for μ and Δ_homo–lumo_, where the pairwise approach did not outperform the pointwise method.

**Table 5:** SchNet Model Performance in the QM7x Data Set^a^

property	MAE	nDCG	Spearmann	accuracy
*E*_PBE0_	28.205 ± 0.22	0.29 ± 0.00	0.22 ± 0.01	0.002
μ	21.118 ± 0.23	0.432 ± 0.01	0.538 ± 0.01	0.11
Δ_homo–lumo_	9.004 ± 0.11	0.896 ± 0.01	0.9 ± 0.00	0.71

a The Schnet was trained using 6 interaction modules and 128 neurons.

The SchNet model exhibited slightly better performance for Δ_homo–lumo_ according to the MAE (Table 1) and Spearman correlation (Table 4) metrics, outperforming both our pairwise and pointwise implementations. However, for the properties *E*_PBE0_ and μ, the pairwise model achieved superior results, particularly for *E*_PBE0_, where its performance was significantly higher. We also observe that when comparing both pointwise models, our pointwise implementation yielded better results for the dipole property μ and *E*_PBE0_.

The performance differences of our implementation can be attributed to key architectural differences. Although our model is based on SchNet’s continuous-filter convolutions, it incorporates skip connections. These connections link features from different network depths directly to the prediction layer, allowing the model to determine more effectively which information is most relevant for estimating a given molecular property.

### 4.2 Evaluation Using the QO2Mol Data Set

The training set for QO2Mol has more than 500 × 10^3^ unique InChIKeys. To train the pairwise model, we selected only groups of InChIKeys with more than 5 configurations. Then, after the first filtering step, we randomly selected 43 × 10^3^ groups of InChIKeys and, within each group, we randomly selected up to 11 molecular configurations to create the pairs for model training. The training pairs are built considering molecular configurations within the same InChIKey group.

For the pointwise training, we randomly selected 54 × 10^3^ groups (InChIKey) and, within each group, we randomly selected up to 11 molecular configurations. The test data set has 28 × 10^3^ groups, which also contain up to 11 randomly selected configurations. As described earlier, train and test sets have distinct InChIKeys and the metrics reported were calculated using the test set. The models were trained for up to 60 epochs. The total sample size for training is approximately 360 × 10^3^, which is much larger than the QM9 data set that has around 130 × 10^3^ molecules and is widely used as a benchmark in many applications of ML.

The results of the ranking performance on the QO2Mol data set are presented in Table 6. The data clearly indicate that the pairwise classifier trained with Entropy Loss is the superior model, achieving the highest performance on all evaluated metrics. The pairwise regression model (Squared Loss) also performed reasonably well but was less effective than the classifier. In contrast, the Pointwise and SchNet models demonstrated considerably weaker performance. Their Spearman correlation coefficients, in particular, were close to zero, which suggests a failure to correctly capture the relative ranking of the items.

**Table 6:** Model Comparison in the QO2Mol Data Set^a^ ^b^ ^c^

metric	entropy loss	squared loss	pointwise	SchNet
MAE	0.862 ± 0.01	1.322 ± 0.01	2.855 ± 0.02	2.985 ± 0.02
nDCG	0.865 ± 0.00	0.78 ± 0.00	0.579 ± 0.00	0.56 ± 0.00
Spearmann	0.828 ± 0.00	0.684 ± 0.01	0.056 ± 0.01	–0.008 ± 0.01
accuracy	0.618	0.457	0.191	0.172

a The Schnet was trained using 6 interaction modules and 128 neurons.

b Squared loss: pairwise regression model.

c Entropy loss: pairwise classifier.

### 4.3 Evaluation on Pretrained Uni-Mol Features

We evaluated the pairwise ranking framework using Uni-Mol models ranging from 84 M to 1.1 B parameters to assess whether the improvements in pairwise training observed with SchNet extend to large-scale pretrained representations. This evaluation focused on the QO2Mol data set as well as the properties *E*_PBE0_ and Δ_homo–lumo_ of QM7X. Tables 7, 8, and 9 show the performance of the model in the QO2Mol data set and the *E*_PBE0_ and Δ_homo–lumo_ properties of the QM7X data set, respectively. We observe that the pairwise approach consistently outperforms the pointwise approach, corroborating the results obtained with the SchNet architecture.

**Table 7:** MLP Model Performance in the QO2Mol Data Set^a^

		MAE	nDCG	Spearmann	accuracy
V1 & 1.1 B	pairwise entropy	2.25 ± 0.01	0.661 ± 0.00	0.347 ± 0.01	0.232
	pairwise MSE	2.412 ± 0.02	0.635 ± 0.00	0.279 ± 0.01	0.201
	pointwise	2.98 ± 0.02	0.573 ± 0.00	0.005 ± 0.01	0.13
V2 & 1.1 B	pairwise entropy	2.835 ± 0.02	0.597 ± 0.00	0.078 ± 0.01	0.157
	pairwise MSE	3.011 ± 0.02	0.585 ± 0.00	0.002 ± 0.01	0.144
	pointwise	3.019 ± 0.02	0.575 ± 0.00	–0.008 ± 0.01	0.134
V1 & 570 M	pairwise entropy	2.255 ± 0.01	0.659 ± 0.00	0.346 ± 0.01	0.227
	pairwise MSE	2.452 ± 0.02	0.632 ± 0.00	0.261 ± 0.01	0.198
	pointwise	3.003 ± 0.02	0.573 ± 0.00	–0.003 ± 0.01	0.129
V2 & 570 M	pairwise entropy	2.899 ± 0.02	0.587 ± 0.00	0.048 ± 0.01	0.148
	pairwise MSE	3.013 ± 0.02	0.585 ± 0.00	0.0 ± 0.00	0.147
	pointwise	3.007 ± 0.02	0.586 ± 0.00	0.003 ± 0.01	0.146
V1 & 310 M	pairwise entropy	2.257 ± 0.01	0.659 ± 0.00	0.345 ± 0.01	0.228
	pairwise MSE	2.385 ± 0.02	0.64 ± 0.00	0.291 ± 0.01	0.208
	pointwise	2.976 ± 0.02	0.575 ± 0.00	0.009 ± 0.01	0.131
V2 & 310 M	pairwise entropy	2.873 ± 0.02	0.589 ± 0.00	0.054 ± 0.01	0.149
	pairwise MSE	3.041 ± 0.02	0.58 ± 0.00	–0.015 ± 0.01	0.139
	pointwise	2.97 ± 0.02	0.594 ± 0.00	0.02 ± 0.01	0.16
V1 & 164 M	pairwise entropy	2.262 ± 0.01	0.657 ± 0.00	0.344 ± 0.01	0.226
	pairwise MSE	2.418 ± 0.02	0.636 ± 0.00	0.274 ± 0.01	0.202
	pointwise	2.971 ± 0.02	0.575 ± 0.00	0.008 ± 0.01	0.131
V2 & 164 M	pairwise entropy	2.838 ± 0.02	0.591 ± 0.00	0.075 ± 0.01	0.148
	pairwise MSE	2.745 ± 0.02	0.612 ± 0.00	0.121 ± 0.01	0.178
	pointwise	3.014 ± 0.02	0.584 ± 0.00	–0.001 ± 0.01	0.145
V1 & 84 M	pairwise entropy	2.244 ± 0.01	0.662 ± 0.00	0.349 ± 0.01	0.233
	pairwise MSE	2.396 ± 0.02	0.639 ± 0.00	0.287 ± 0.01	0.205
	pointwise	3.004 ± 0.02	0.573 ± 0.00	–0.002 ± 0.01	0.132
V2 & 84 M	pairwise entropy	2.733 ± 0.02	0.611 ± 0.00	0.116 ± 0.01	0.179
	pairwise MSE	2.884 ± 0.02	0.596 ± 0.00	0.056 ± 0.01	0.155
	pointwise	2.955 ± 0.02	0.578 ± 0.00	0.012 ± 0.01	0.136

a Ranking performance comparison for the unimol library in the QO2Mol data set.

**Table 8:** MLP Model Performance in the QM7X Data Set for *E*_PBE0_ ^a^

		MAE	nDCG	Spearmann	accuracy
V1 & 1.1 B	pairwise entropy	9.424 ± 0.09	0.887 ± 0.01	0.904 ± 0.00	0.874
	pairwise MSE	10.109 ± 0.1	0.734 ± 0.01	0.887 ± 0.00	0.548
	pointwise	22.515 ± 0.24	0.38 ± 0.01	0.5 ± 0.01	0.073
V2 & 1.1 B	pairwise entropy	33.48 ± 0.15	0.278 ± 0.00	0.007 ± 0.01	0.003
	pairwise MSE	33.636 ± 0.19	0.277 ± 0.00	0.001 ± 0.01	0.007
	pointwise	32.999 ± 0.29	0.266 ± 0.00	0.023 ± 0.01	0.003
V1 & 570 M	pairwise entropy	9.656 ± 0.08	0.865 ± 0.01	0.901 ± 0.00	0.819
	pairwise MSE	10.193 ± 0.1	0.737 ± 0.01	0.885 ± 0.00	0.548
	pointwise	26.955 ± 0.25	0.342 ± 0.01	0.314 ± 0.01	0.04
V2 & 570 M	pairwise entropy	33.495 ± 0.12	0.276 ± 0.00	0.007 ± 0.01	0.01
	pairwise MSE	33.777 ± 0.11	0.277 ± 0.00	–0.006 ± 0.01	0.004
	pointwise	32.709 ± 0.26	0.273 ± 0.00	0.042 ± 0.01	0.009
V1 & 310 M	pairwise entropy	9.455 ± 0.09	0.875 ± 0.01	0.903 ± 0.00	0.847
	pairwise MSE	11.058 ± 0.1	0.628 ± 0.01	0.866 ± 0.00	0.345
	pointwise	32.388 ± 0.27	0.262 ± 0.00	0.056 ± 0.01	0.002
V2 & 310 M	pairwise entropy	32.103 ± 0.3	0.271 ± 0.00	0.066 ± 0.01	0.005
	pairwise MSE	33.849 ± 0.19	0.273 ± 0.00	–0.012 ± 0.01	0.009
	pointwise	33.554 ± 0.11	0.278 ± 0.00	0.004 ± 0.01	0.008
V1 & 164 M	pairwise entropy	9.656 ± 0.09	0.864 ± 0.01	0.9 ± 0.00	0.815
	pairwise MSE	10.322 ± 0.1	0.735 ± 0.01	0.883 ± 0.00	0.553
	pointwise	28.563 ± 0.29	0.296 ± 0.00	0.24 ± 0.01	0.014
V2 & 164 M	pairwise entropy	32.48 ± 0.27	0.272 ± 0.00	0.049 ± 0.01	0.004
	pairwise MSE	34.225 ± 0.18	0.275 ± 0.00	–0.033 ± 0.01	0.007
	pointwise	33.531 ± 0.15	0.279 ± 0.00	0.006 ± 0.01	0.01
V1 & 84 M	pairwise entropy	9.4 ± 0.08	0.88 ± 0.01	0.905 ± 0.00	0.854
	pairwise MSE	10.03 ± 0.1	0.742 ± 0.01	0.89 ± 0.00	0.551
	pointwise	34.351 ± 0.23	0.27 ± 0.00	–0.04 ± 0.01	0.005
V2 & 84 M	pairwise entropy	35.195 ± 0.29	0.256 ± 0.00	–0.088 ± 0.01	0.002
	pairwise MSE	33.928 ± 0.34	0.267 ± 0.00	–0.038 ± 0.07	0.004
	pointwise	31.289 ± 0.25	0.281 ± 0.00	0.109 ± 0.01	0.009

a Ranking performance comparison for the Uni-Mol library in the QM7X data set.

**Table 9:** MLP Model Performance in the QM7X Dataset for Δ_homo–lumo_ ^a^

		MAE	nDCG	Spearmann	accuracy
V1 & 1.1 B	pairwise entropy	18.11 ± 0.16	0.59 ± 0.01	0.666 ± 0.01	0.221
	pairwise MSE	18.812 ± 0.17	0.673 ± 0.01	0.637 ± 0.01	0.343
	pointwise	30.762 ± 0.29	0.347 ± 0.01	0.134 ± 0.01	0.053
V2 & 1.1 B	pairwise entropy	33.695 ± 0.11	0.282 ± 0.00	–0.004 ± 0.01	0.011
	pairwise MSE	33.718 ± 0.11	0.281 ± 0.00	–0.004 ± 0.01	0.014
	pointwise	33.062 ± 0.24	0.316 ± 0.01	0.02 ± 0.01	0.04
V1 & 570 M	pairwise entropy	18.356 ± 0.16	0.579 ± 0.01	0.658 ± 0.01	0.202
	pairwise MSE	18.708 ± 0.17	0.672 ± 0.01	0.64 ± 0.01	0.332
	pointwise	25.712 ± 0.19	0.482 ± 0.01	0.362 ± 0.01	0.136
V2 & 570 M	pairwise entropy	32.629 ± 0.24	0.326 ± 0.01	0.039 ± 0.01	0.045
	pairwise MSE	33.822 ± 0.11	0.28 ± 0.00	–0.009 ± 0.01	0.012
	pointwise	34.523 ± 0.25	0.297 ± 0.01	–0.057 ± 0.01	0.03
V1 & 310 M	pairwise entropy	18.205 ± 0.16	0.585 ± 0.01	0.662 ± y0.01	0.206
	pairwise MSE	18.778 ± 0.17	0.681 ± 0.01	0.638 ± 0.01	0.355
	pointwise	28.904 ± 0.21	0.381 ± 0.09	0.221 ± 0.01	0.071
V2 & 310 M	pairwise entropy	33.197 ± 0.22	0.307 ± 0.01	0.015 ± 0.01	0.027
	pairwise MSE	33.378 ± 0.21	0.319 ± 0.01	0.008 ± 0.01	0.045
	pointwise	35.231 ± 0.25	0.296 ± 0.01	–0.091 ± 0.01	0.036
V1 & 164 M	pairwise entropy	18.139 ± 0.16	0.574 ± 0.01	0.666 ± 0.01	0.197
	pairwise MSE	18.817 ± 0.17	0.678 ± 0.01	0.637 ± 0.01	0.352
	pointwise	29.137 ± 0.21	0.375 ± 0.01	0.212 ± 0.01	0.067
V2 & 164 M	pairwise entropy	33.849 ± 0.18	0.288 ± 0.00	–0.016 ± 0.01	0.017
	pairwise MSE	33.426 ± 0.21	0.299 ± 0.01	0.002 ± 0.01	0.029
	pointwise	35.438 ± 0.26	0.286 ± 0.01	–0.103 ± 0.01	0.026
V1 & 84 M	pairwise entropy	18.533 ± 0.16	0.575 ± 0.01	0.651 ± 0.01	0.199
	pairwise MSE	18.702 ± 0.17	0.677 ± 0.01	0.641 ± 0.01	0.354
	pointwise	30.052 ± 0.21	0.344 ± 0.01	0.171 ± 0.01	0.041
V2 & 84 M	pairwise entropy	33.531 ± 0.26	0.322 ± 0.01	–0.007 ± 0.01	0.044
	pairwise MSE	35.928 ± 0.25	0.287 ± 0.01	–0.129 ± 0.01	0.024
	pointwise	34.564 ± 0.26	0.302 ± 0.01	–0.058 ± 0.01	0.031

a Ranking performance comparison for the Uni-Mol library in the QM7X data set.

Figures 6 and 7 compare the MAE and nDCG, respectively, for pairwise approaches considering the property *E*_PBE0_. A key observation is that the features provided by the V1 version of Uni-Mol are significantly more effective for the ranking task than those of V2. This empirical result supports the hypothesis that V2’s equilibrium-focused optimization tends to smooth out the specific geometric distortions of nonequilibrium conformers (see Section 2.3), effectively collapsing the variance needed for ranking. In contrast, V1’s denoising objective preserves these subtle geometric distinctions. Moreover, for the V1 embeddings, the pairwise approach using the cross-entropy loss function yielded superior results compared to the squared loss.

**Figure 6:**
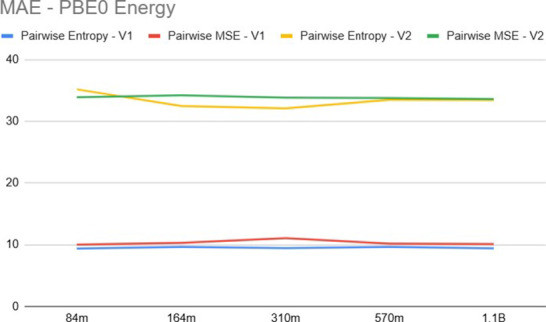
Ranking mean absolute error for *E*_PBE0_.

**Figure 7:**
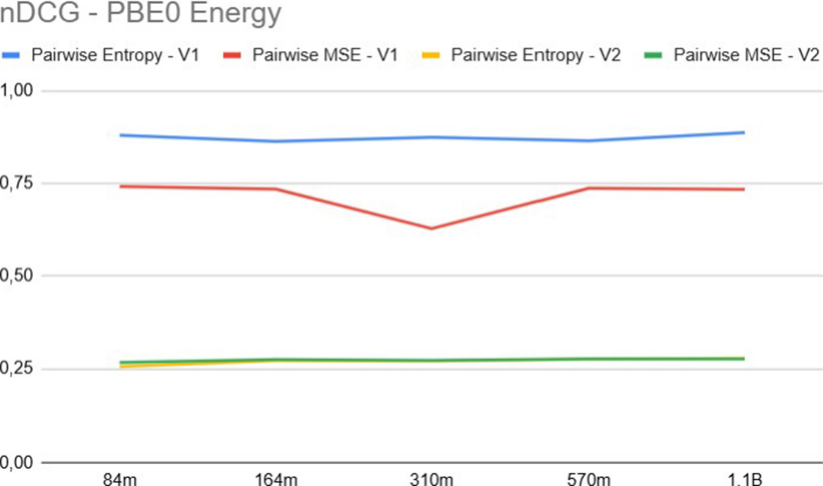
nDCG for energy *E*_PBE0_.

Figures 8 and 9 present the results for the Δ_homo–lumo_ property across different model sizes. Here, we observe that for the V1 embedding, the MAE is higher than that for *E*_PBE0_, a trend also noted in Table 1. While Table 1 showed the pointwise approach achieving a lower MAE for this specific property, the V1 features still consistently outperform V2. Finally, observing Figure 6 through Figure 9, we notice that increasing model size yields diminishing returns, with performance saturating rapidly; for instance, the 84 M model often achieves competitive results compared to the 1.1 B variant, suggesting that the pretraining objective (V1 vs V2) is more critical than model capacity for this ranking task.

**Figure 8:**
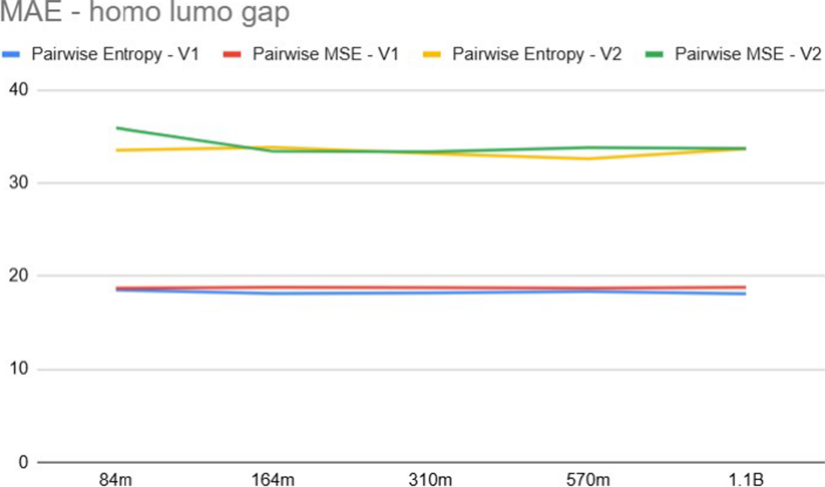
Ranking mean absolute error for Δ_homo–lumo_.

**Figure 9:**
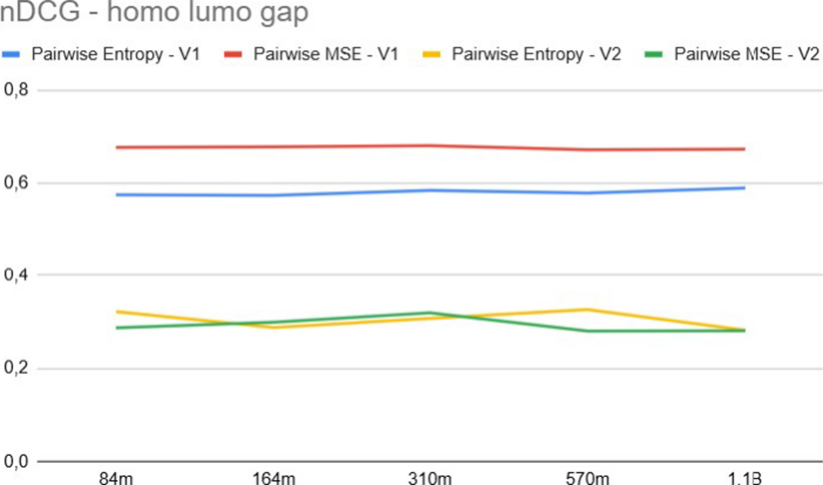
nDCG for Δ_homo–lumo_.

## 5 Conclusions

In this study, we explored the use of two distinct approaches for ranking molecules based on their properties, with implications for the screening of molecules or molecular structures. Notably, our method differs from traditional deep learning approaches by utilizing nonequilibrium structures to train the model, enabling it to learn how to rank molecules based on specific properties. This ranking can be used, for instance, to select a subset composed of top-ranked structures having their properties computed by DFT.

We compared two approaches: point-wise model training and pairwise model training. For the pairwise models, we trained two different models: the first using a binary classification loss function and the second using a squared loss function. The pairwise approach was inspired by the work of Köppel et al.,^26^ where the model learns during training to identify the molecular structure of the pair with the highest property value using either pairwise classification or difference regression. We evaluated the models using four different metrics, including MAE, Spearman correlation, nDCG, and the concordance of the top-ranked molecule with the smallest property value. Our results indicate that the pairwise method looks promising for ranking molecular structures. This conclusion is reinforced by additional experiments with the Uni-Mol Transformer architecture, where pairwise training consistently outperformed pointwise regression across different model sizes and data sets. These findings suggest that the benefits of pairwise ranking are architecture-agnostic and can enhance the screening capabilities of both bespoke GNNs and large-scale pretrained models.

The pairwise methods (Entropy and Squared Loss) excel at predicting total energies and their direct additive components. The pairwise method has an advantage over the pointwise approach, especially when molecular configurations have very similar energies. A pointwise model may struggle in these cases as it evaluates each structure independently. In contrast, a pairwise model directly compares pairs of structures and their energy differences. This allows it to discern subtle distinctions, as long as the input features for each structure differ. Consequently, the pairwise framework is optimized to learn a scoring function based on these relative differences, enabling it to more effectively determine the correct rank ordering. However, in QM7x, the pointwise method wins on properties that are calculated from differences (homolumo gap and atomization energy), and dipole moment.

Future work can focus on building on our findings and exploring the full potential of the pairwise ranking approach. Specifically, it will be interesting to investigate the underlying reasons for the struggles of the pairwise method with certain properties and to develop targeted strategies to address these challenges. Another possibility is to investigate other ranking approaches besides pairwise ranking, such as list-wise ranking, or to apply our pairwise framework to more sophisticated architectures beyond our current SchNet-based model. Furthermore, it can provide valuable insights for model architecture development in order to investigate which molecules the model has the most difficulty predicting. The presence of a chemical group may make the molecular property more difficult to learn from a Machine Learning point of view. Last but not least, it would be interesting to improve the evaluation with more data sets.

## Supplementary Material



## Data Availability

The source code for the model can be found in the github repository.^41^

## Notes

More details about model training, data split, and performance metrics (PDF)
