# Supplementary material for: Pairwise Neural Networks for Ranking Molecular Structures Based on Properties
Source: ACS Omega. 2026 Feb 12;11(7):12852–64. doi: 10.1021/acsomega.6c00717 (PMC12947046; doi:10.1021/acsomega.6c00717)
Supplement: Supplementary file 1 [file ao6c00717_si_001.pdf]

Pairwise Neural Networks for Ranking Molecular Structures Based on Properties

Renato Frazzato Viana,*,† Juarez L. F. Da Silva,*,‡ Luis G. Dias,*,¶ and Ronaldo C. Prati*,†

†*Center of Mathematics, Computation and Cognition, Federal University of ABC, Av. dos Estados, 5001,* 09210-580*, Santo André, SP, Brazil*

‡*São Carlos Institute of Chemistry, University of São Paulo, Av. Trabalhador São-Carlense* 400*,* 13560-970*, São Carlos, SP, Brazil*

¶*Chemistry Department, FFCLRP, University of São Paulo,* 14040-901*, Ribeirão Preto, SP, Brasil*

E-mail: renatofrazzatoviana@gmail.com; juarez_dasilva@iqsc.usp.br; lgdias@ffclrp.usp.br; ronaldo.prati@ufabc.edu.br

Contents

S-1 Introduction **S-2**

S-2 Definition of Performance Metrics **S-2**

S-2.1 MAE - Mean Absolute Error S-2

S-2.2 nDCG - Normalized Discounted Cumulative Gain S-3

S-2.3 Lowest Energy Structure Classification S-3

S-2.4 Spearman's rank coefficient S-4

S-3 Model Training **S-4**

References **S-6**

S-1 Introduction

The supporting information file presented here provides additional technical details that augment and substantiate the replication of our proposed methodology.

S-2 Definition of Performance Metrics

In this section, we define the metrics used to evaluate the model's performance. We employed four metrics: MAE (mean absolute error), nDCG, Spearman correlation, and accuracy in classifying the structure with the lowest energy value.

The dataset consists of multiple molecules, each with associated isomers and conformers. In other words, it is composed of triplets $(\text{molecule}, \text{isomer}, \text{conformer})$. For each triplet in the test dataset, we rank the structures based on the model's predictions and compare them to the rankings derived from the true property values (i.e., the target property the model was trained on).

S-2.1 MAE - Mean Absolute Error

The motivation for this metric is to quantify how much the model's ranking ($\hat{r}$) deviates from the true ranking (r). For each molecule, isomer, and conformer (mic), for the dataset providing K structures (s), where we evaluate the ranking accuracy.

$$mae_{mic} = \frac{1}{K} \sum_{s=1}^{K} |r_s - \hat{r}_s|$$

After calculating the values mae_{mic} we aggregate them averaging.

$$MAE = \frac{1}{T} \sum mae_{mic}$$

where T is the total of isomers and conformers of molecules in the test dataset.

S-2.2 nDCG - Normalized Discounted Cumulative Gain

Normalized Discounted Cumulative Gain[1] is very popular in model comparison for ranking context. The idea of this metric is compare the gain obtained by the model's rank and the gain obtained by the ideal rank. This metric is a percentage ranging from 0% to 100%.

The discounted cumulative gain is defined as:

$$dcg_{mic} = \sum_{s=1}^{K} \frac{relevance(s)}{1 + log(r_s)}$$

Where r_s represents the molecular structure rank and relevance(s)=exp$(-r_s^*)$, here we use r_s^* to represent the true ranking and we can observe that when rank increases the relevance of the structure decreases. The dcg_{mic} penalizes the relevance of the structure considering the rank positions, i.e, if the structure has a lower position in the rank the contribution of the relevance in the final dcg will be diminished. When the dcg is calculated considering the true ranking we obtain the ideal dcg(idcg) which represents the maximum value possible for dcg because the true ranking provides the minimum penalization to the structures relevance. Hence, dcg is calculated considering the rank provided by the model and considering the true ranking(idcg) and the ndcg is given by the ratio.

$$ndcg_{mic} = \frac{dcg_{mic}}{idcg_{mic}}$$

The final nDCG is given by the average across all $ndcg_{mic}$.

$$nDCG = \frac{1}{T} \sum ndcg_{mic}$$

S-2.3 Lowest Energy Structure Classification

Identifying molecular structures with low energy is essential in several scientific and industrial applications. Some applications in material science include: the physical properties of materials depend on their atomic and molecular structure. The most stable (lowest energy) configurations determine mechanical strength, conductivity, and other properties. Designing

advanced materials like graphene, perovskites, and metal-organic frameworks (MOFs) for applications in semiconductors, batteries, and catalysts. Applications in pharmacology may include low-energy conformations of molecules are more stable and likely to represent the bioactive form when binding to a target protein.

This metric is calculated across each triplet $(\text{molecule}, \text{isomer}, \text{conformer})$. If the model is able to place the lowest energy in first position then 1 is added to a counter else 0 is added then, the sum is divided by the total number of molecules, isomers and conformers.

S-2.4 Spearman's rank coefficient

Spearman's correlation is widely used in various fields where the relationship between two variables is monotonic but not necessarily linear, this coefficient is also very popular in ranking measurement. The Spearman coefficient can be calculated in a following way.

Considering two variables X and Y, let rank(X) and rank(Y) be the rank provided by those variables. The expression for Spearman coefficient is:

$$\rho = 1 - \frac{6\sum d_i^2}{n(n^2-1)}$$

where d_i = rank(X_i) - rank(Y_i). Interesting relation between Spearman and Pearson correlation is that Spearman can be obtained applying Pearson on ranks, i.e, Spearman = Pearson(rank(X), rank(Y)). In our scenario, i represents the molecular structure and d_i is the difference of the ranks provided by model and the true rank.

We calculated the Spearman coefficient in the same way we did for the previous metrics. For each triplet $(\text{molecule}, \text{isomer}, \text{conformer})$, we compared the rank provided by the model vs the true rank by the target property. Then, we average across all the Spearman values.

S-3 Model Training

The QM7-X training dataset comprises 6591 molecules, with each molecule exhibiting an average of six isomers and conformers.[2] For each triplet $(\text{molecule}, \text{isomer}, \text{conformer})$, there are 101 configurations (structures) available. The average molecular size is 17 atoms, which

incorporate the following chemical elements: C, Cl, H, N, O, S. This dataset contains ≈ 4.2 million of structures of small organic molecules. From this extensive collection of molecules, 359 molecules have been selected for the testing dataset. In addition, the training dataset is segregated into two segments: one designated for model training and the other for validation, with 10 % of the 6591 molecules apportioned for validation.

The pairwise model does not require much more effort than the pointwise. The main bottleneck is the pair generation for example, in the pointwise approach the molecular structures flow into the model independently. In the pairwise it is necessary to provide pairs however, considering a certain molecule has n configurations then we would need to generate n!/(2!(n-2)!) pairs in order to provide all possible combinations to the model. For example, certain isomer and conformer has 101 configurations then to explore all the pairs this means generate 5050 pairs.

In order to avoid such an effort and accelerate learn-to-rank training we made an approximation, we randomly select 30 configurations from each triplet $(\text{molecule}, \text{isomer}, \text{conformer})$. In addition, training batches are randomly generated, comprising 64 pairs sampled from each triplet. We sample(with replacement) two times 64 structures, then the molecular structure in the first position of the first sample will be matched with the first position of the second sample and so on. For the training in QO2Mol[3], in each InChIKey we did the same sampling approach but the sample size is eleven. In QO2Mol dataset, each InChIKey has different amount of molecular configurations so, we randomly selected up to eleven and to generate the pairs for training, for each InChIKey we sampled with replacement twice all the structures.

The models were implemented in Tensorflow and the pairwise models were trained considering two different loss function, cross-entropy and squared regression loss up to 60 epochs and they were trained using Google Colab environment in a L4 and A-100 GPU hardware.

We also trained a pointwise regression model to compare to pairwise ranking ability. For pointwise model training we also used batch size of 64 molecules structures and trained up to 60 epochs. Apart from that, to expedite the training we sampled randomly 100 structures for each molecule, i.e, sample of 100 structures independently of isomers and conformers.

References

1 Järvelin, K.; Kekäläinen, J. Cumulated gain-based evaluation of IR techniques. *ACM Transactions on Information Systems (TOIS)* **2002**, *20*, 422–446.

2 Hoja, J.; Sandonas, L. M.; Ernst, B. G.; Vazquez-Mayagoitia, A.; DiStasio Jr, R. A.; Tkatchenko, A. QM7-X, a comprehensive dataset of quantum-mechanical properties spanning the chemical space of small organic molecules. *Scientific data* **2021**, *8*, 1–11.

3 Liu, W.; Ai, X.; Zhou, Z.; Qu, C.; An, J.; Zhou, Z.; Cheng, Y.; Xu, Y.; Cao, F.; Qi, A. An Open Quantum Chemistry Property Database of 120 Kilo Molecules with 20 Million Conformers. *arXiv preprint arXiv:2410.19316* **2024**,
